# Using new cold chain technologies to extend the vaccine cold chain in India: Equipment performance, acceptability, systems fit, and costs

**DOI:** 10.1016/j.jvacx.2023.100385

**Published:** 2023-09-09

**Authors:** Sandeep Kumar, Mercy Mvundura, Arindam Ray, Pradeep Haldar, Pat Lennon, Nancy Muller, Arup Deb Roy, Sachin Rewaria

**Affiliations:** a PATH, New Delhi, India; b PATH, Seattle, USA; c Bill & Melinda Gates Foundation, India Country Office, New Delhi, India; d Government of India, Ministry of Health and Family Welfare, Delhi, India; e Immunisation Technical Support Unit, Government of India, Delhi, India

**Keywords:** Vaccine cold chain, Cold chain equipment, Second-generation ice-lined refrigerator, Solar direct drive refrigerator, Long-term passive device, Immunisation

## Abstract

This study evaluated the performance, acceptability, costs, and systems fit of three new cold chain devices in India: a second-generation ice-lined refrigerator (ILR), a solar direct drive (SDD) refrigerator, and a long-term passive device (LTPD). The evaluation was conducted over 15 months during 2016–2017. Sites were selected for their diversity in climate, terrain, and grid electrical supply, and 31 cold chain devices were deployed, 1 to each site. Results showed that all three technologies maintained correct temperatures. The SDD refrigerators had no malfunctions, whilst the ILRs had at least one malfunction, mostly due to the printed circuit board’s sensitivity to the erratic power supply. The LTPD temperature display panel caused challenges initially that required replacement of all solar panels with lithium batteries. Yet the devices’ long holdovers helped ensure vaccine potency. One challenge, particularly with the ILRs and SDD refrigerators, was condensation. The passively cooled LTPD was valued in settings with smaller populations and unreliable or no power; however, some its features, including the need to condition ice blocks, made it challenging to operate. In addition, the acceptable temperature range for the LTPD, as for all passively cooled devices (greater than 0 °C and less than + 10 °C), was confusing for some health workers due to the decades-long emphasis on maintaining temperatures at + 2 °C to + 8 °C. The greatest system-related benefit was establishment of new cold chain points (CCPs) at locations with intermittent or no grid electricity, bringing immunisation services closer to hard-to-reach areas. A key limitation of all three devices was the inability to freeze ice packs, which are required for vaccine carriers, somewhat restricting the potential of these technologies to reach underserved populations. Moreover, establishing new CCPs added costs to the health system. Results from this study, including costing data, can help guide decision-making.

## Highlights

• The study evaluated three new cold chain devices in India: electric, solar, passive.
• All devices maintained correct temperatures but with a few excursions and failures.
• A major benefit was helping establish new cold chain points in hard-to-reach areas.
• Conditioning of ice blocks made the passive cooling device challenging to operate.
• A key limitation for each device: the inability to freeze ice for vaccine carriers.

## Introduction

India’s population of 1.3 billion [1] is spread over 3.2 million square kilometers [2], with extremes not only in climate and temperature but also in population density. India’s Universal Immunisation Programme is one of the largest immunisation programmes globally in terms of the quantity of doses delivered, number of beneficiaries reached, number of immunisation sessions organised, geographical spread, and diversity of areas covered. The programme targets 27 million newborns and 30 million pregnant women each year. This requires more than 150 million doses of vaccines [3]. Spanning this diversity is the country’s cold chain network, the backbone of its immunisation programme.

Vaccines save lives and reduce the risk of infection and disease transmission by strengthening the body’s immune system. Immunisation prevents disease outbreaks. Currently, 3.5 to 5 million deaths per year are prevented from early childhood immunisations [4]. Vaccines are highly thermosensitive biological substances that have a fixed shelf life and lose potency over time. This loss of potency is irreversible and accelerated if proper storage and temperature conditions are not maintained. Therefore, it is important for every country to have a robust immunisation cold chain from the national level to the point of vaccination. The cold chain is a defined set of standards, procedures, facilities, and equipment intended to maintain safe storage and transport of vaccines in the appropriate temperature range from the manufacturer to the vaccine recipient. This ensures that safe and potent vaccines are delivered to beneficiaries.

Despite an increase in the number of cold chain points (CCPs), the network has not kept up with India’s population growth [5]. India’s more than 27,000 CCPs serve an average population of about 46,000 each [3]; and in some states, a CCP can serve more than 150,000 [6]. The biggest challenge, however, is not the supply of vaccines but rather the safe storage of vaccines at remote locations [6]. In addition, the availability and reliability of electricity is highly variable from district to district, especially at the village level, and health facilities/CCPs rely on generators to provide back-up power when electricity supply is erratic [3]. These issues present a direct challenge to expanding the cold chain network to be as close as possible to session sites; and in this environment, electric-powered cold chain equipment that has longer holdover times or non-electric-powered cold chain equipment may be optimal.

Of the 27,000 CCPs, 1,332 have less than 8 h of electricity supply within a 24-hour period [6]. Adequate and appropriate cold chain equipment that increases equitable access to immunisation services is required to reach even the most inaccessible areas.

Many health facilities and CCPs, especially those located at the village level, have limited or erratic electrical supply, creating a reliance on generators for back-up power. This situation presents an obstacle to expanding the cold chain network because existing equipment models need consistent grid electricity to properly function [7], [8], [9], [10]. Alternative cold chain equipment is required that can ensure safe storage and delivery of vaccines in areas that are geographically isolated or sparsely populated, are subject to extremes in temperature, or lack reliable access to electricity.

The Piloting New Cold Chain Technologies in India project evaluated the ability of three innovative devices identified by PATH and the Immunisation Technical Support Unit of the Ministry of Health and Family Welfare (MoHFW) to overcome these challenges.

## Materials and methods

### Cold chain equipment

The devices evaluated were a second-generation ice-lined refrigerator (ILR), the Godrej & Boyce MFG. Co. Ltd., GVR 100 AC (Sure Chill®); a solar direct drive (SDD) refrigerator, the Godrej & Boyce MFG. Co. Ltd., GVR 100 DC (Sure Chill®); and a long-term passive device (LTPD), the AUCMA Co. Ltd., ARKTEK™ model YBC-5 (P6). All of these devices are approved by the World Health Organization’s (WHO’s) Performance, Quality and Safety (PQS) programme [11].

**Fig. 1:**
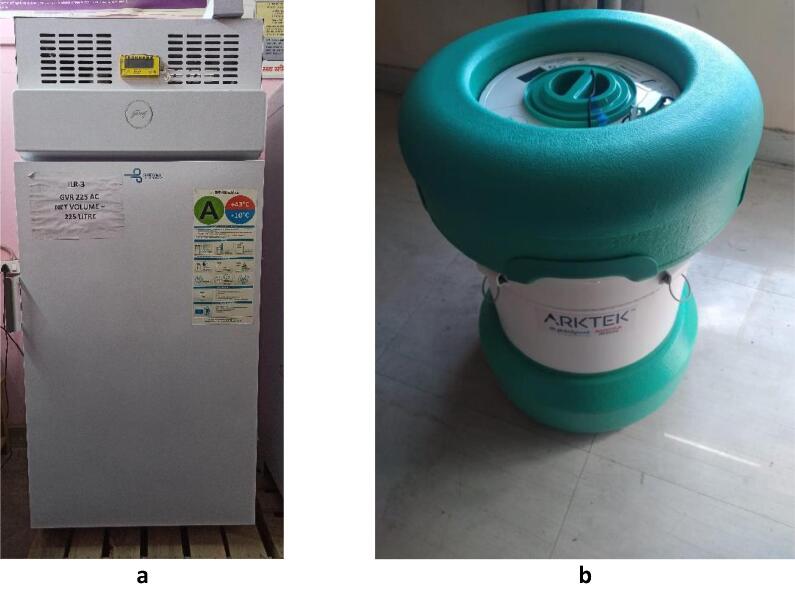
The three devices evaluated as part of the study: (a) ice-lined refrigerator and solar direct drive refrigerator, (b) long-term passive device.

Note: the ice-lined refrigerator and solar direct drive refrigerator look the same except that the solar direct drive refrigerator has solar panels installed on the rooftop.

The three devices (see Fig. 1) were identified by the project team in consultation with the MoHFW Immunisation Division based on the proposed benefits (long holdover period for vaccine storage and establishment of new CCPs). A pilot study was proposed to assess equipment performance, acceptability, systems fit, and cost, given the potential benefits. These evaluation criteria were developed using WHO’s generic guide for field evaluations [12].

### Study duration, site selection, and equipment distribution

The evaluation was conducted over 15 months, from March 2016 through May 2017, in four states in India: Rajasthan, Uttar Pradesh, Meghalaya, and Uttarakhand. The Immunisation Technical Support Unit managed project implementation in Rajasthan and Uttarakhand. PATH managed implementation in Uttar Pradesh and Meghalaya. Four pilot districts were selected, one per state. Site selection criteria included areas with variations in population density, reliability of electrical supply, terrain (flat or hilly), and seasonal temperature extremes (Table 1). For the purpose of data collection, we divided the year into three seasons: summer (March through May), monsoon (June through October), and winter (November through February).

**Table 1:** Site characteristics per selection criteria.*

**State**	**District**	**Criteria**	Density (people per km^2^)*
**Rajasthan**	Jaisalmer	Tropical climate with extreme high temperatures in summer; temperature variations during day and night.	17
**Uttar Pradesh**	Kushinagar	Tropical climate with high temperatures in summer; health facilities with minimal electricity (3 to 5 h) catering to large populations.	1,227
**Meghalaya**	Jaintia Hills	Temperate climate with high rainfall and humidity; difficult terrains.	103
**Uttarakhand**	Tehri Garhwal	Temperate climate with extreme cold temperatures in winter; hard-to-reach areas.	170

* Population density from: Census Organisation of India. *Population Census 2011*. New Delhi: India; 2020. Available at: https://www.census2011.co.in/ [accessed 05 May 2020].

Implementation and data collection occurred in two phases. In Phase 1, placeholder vaccines labeled “Not for Human Use” were stored and transported in the equipment during all three seasons, from early March 2016 through early January 2017. In Phase 2, vaccines were stored, transported, and used for immunisations in winter and summer only, from early January 2017 through end of May 2017.

A total of 31 CCPs took part in the study: 20 were newly established and 11 were existing. The existing CCPs were selected as they catered to large catchment areas and their refrigerators were either old or the CCP had electrical supply issues. All existing CCPs were using ILRs at baseline, and all CCPs received new equipment as part of the study. During Phase 1, one device was installed per CCP: 9 ILRs, 4 SDD refrigerators, and 18 LTPDs. Each state received a mix of device types (Table 2).

**Table 2:** Number of equipment and type installed at selected health facilities in each region.

**Type of device**	**Second-generation ice-lined refrigerator**	**Solar direct drive refrigerator**	**Long-term passive device**
**Characteristics of selected health facilities/cold chain points**	• Less than 8 h of electricity. Catering to a large population or large geographical area.	• No or highly erratic electricity supply. Catering to a large population.	• Minimal (3 to 5 h) electricity. Catering to subcentres or relatively small populations.
**Rajasthan**	**2**	**1**	**5**
**Uttar Pradesh**	**2**	**1**	**3**
**Meghalaya**	**2**	**1**	**6**
**Uttarakhand**	**3**	**1**	**4**
**TOTAL**	**9**	**4**	**18**

**Table 3:** Internal temperature summary for second-generation ice-lined refrigerators.

**State**	**Second-generation ILRs—Phase 1** **n = 116,214 data points total**		**Second-generation ILRs—Phase 2** **n = 58,026 data points total**	
	**Mean (SD)**	**95% CI**	**Mean (SD)**	**95% CI**
**Rajasthan**	5.6 (0.9)	5.58–5.61	5.4 (1.3)	5.36–5.40
**Uttar Pradesh**	5.4 (0.6)	5.34–5.36	5.5 (0.9)	5.47–5.50
**Meghalaya**	4.9 (0.6)	4.85–4.86	5.0 (2.5)	5.00–5.08
**Uttarakhand**	4.7 (0.5)	4.74–4.75	4.6 (0.6)	4.55–4.50

CI: confidence interval; ILR: ice-lined refrigerator; SD: standard deviation.

### Training

In India, cold chain handlers (CCHs) prepare vaccines for auxiliary nurse midwives (ANMs) to transport to immunisation session sites. Each CCH reviews the site demand and prepares the vaccine carrier(s) accordingly. Similarly, after the session(s), all vaccine carriers are brought back to the CCP. CCHs then check the vaccine vial monitor status of each vaccine, verify the opened date, and return viable vaccines to the cold chain equipment. Before Phase 1 implementation, state and district MoHFW officials trained the CCHs according to the government of India’s *Handbook for Vaccine & Cold Chain Handlers* [13]. Equipment manufacturer representatives provided training on each new cold chain technology. A total of 108 participants attended the trainings across the project districts. A refresher training was provided before Phase 2 implementation.

### Evaluation components

The study evaluated the devices’ performance, specifically their ability to maintain temperatures within the correct range in all seasons and with limited or highly erratic power supply. Two measures of air temperature data were collected, one from the device’s vaccine storage compartment and the other from the ambient temperature in the health facility. Data were collected every 30 min over the entire study duration of 15 months using LogTag® TRIX-8, a temperature monitoring device prequalified by WHO PQS for use as a “user programmable temperature data logger” to monitor vaccine storage and transport temperatures. The monitoring device has a manufacturer-quoted “rated absolute accuracy” of ± 0.5 °C within the temperature range –20 °C to + 40 °C.

One LogTag TRIX-8 sensor was placed on a shelf inside each cold chain device to log internal air temperatures, and one was tied to the outside of each cold chain device to log ambient air temperature in the room. Following the WHO field evaluation protocol and temperature monitoring guidelines, we placed the LogTag TRIX-8 internal sensor approximately at the geometric centre of the vaccine storage area, as allowed by shelving and container configuration. The sensor was not secured and was placed on the shelf or in the container so as to avoid contact with the vaccine load, making the locations somewhat variable. The external, ambient sensor was tied with string to a side handle of the LTPD; the sensor hung approximately halfway down the cold chain device. The other cold chain devices each have a front handle, to which a sensor was tied so that it hung approximately a quarter-meter below the bottom of the handle. The external sensors were not in close proximity to any of the thermally active elements of the refrigerators (e.g., compressors, ventilation fans, condensers), but were in light contact with the cold chain device external walls whilst hanging freely.

LogTags measure air temperature and do not directly approximate the thermal properties of vaccines, which are variable based on fill liquid, fill volume, primary packaging type, and location. The TRIX-8 devices are not wire probes and have non-negligible thermal mass. As specified by the manufacturer, they have a temperature reading stabilisation time to register 90% of an immediate step-change in temperature (referred to as T90) of typically less than 5 min in air moving at 1 m/s. LogTag temperature data from all four states were aggregated and analysed using SPSS statistical software. Quantitative data on power supply were collected through an electric power meter; data on humidity were collected using a humidity meter device. Temperature readings during equipment malfunction were excluded from the analysis.

Using data from the project, the study team estimated the incremental costs to the health system of establishing new CCPs. These costs included procurement, installation, operations, repairs (as needed), and training. The team used structured baseline and endline questionnaires to interview health workers at each CCP; these data were used to estimate the costs for time spent travelling to and from outreach sites before and after a new CCP was established. Both financial and economic costs were reported. Financial costs involved transactions with monetary outlays or expenditures, whilst economic costs quantified resources based on opportunity cost, regardless of whether a financial transaction occurred [14]. Only costs for the health system were reported. Qualitative data were collected through individual, in-depth interviews at the end of each phase on the devices’ acceptability to CCHs and ANMs and fit within the Indian health system. The study interviewed 37 respondents, primarily CCHs and medical officers at baseline, and 34 CCHs, 23 ANMs, and 25 beneficiaries at endline.

### Equipment modification

In Phase 1, condensation was observed in the ILRs and SDD refrigerators, mainly on the side walls and top, inside the vaccine storage compartments. To maintain proper storage conditions, the project team and manufacturer proposed installing plastic strip curtains on the units. Strip curtains are one of several potential humidity mitigation approaches. Manufacturers should consider humidity control measures that are effective at preventing condensation in high humidity environments and that do not interfere with user access to vaccines or refrigerator performance. To further protect the vaccine boxes and vial labels from moisture, the vaccines were stored in plastic boxes with lids. Four different configurations were used throughout the four states: with curtains, with plastic boxes; with curtains, without plastic boxes; without curtains, with plastic boxes; and without curtains, without plastic boxes.

## Results

### Performance

The key performance metrics for each device were internal temperature and overall durability.

#### Second-generation ice-lined refrigerator

The ILR offered reliable protection against freezing: temperatures remained at greater than + 4 °C at all times. In this assessment, the ILR confirmed the WHO PQS Grade A freeze protection rating it earned in laboratory settings. The mean internal temperature of the ILR across both phases ranged from + 4.6 °C to + 5.6 °C, well within the desired + 2 °C to + 8 °C range (Table 3).

The ILR maintained temperatures within the correct range 99.6% of the time in Phase 1 and 98% of the time in Phase 2, and the average internal temperature was + 4.9 °C. Internal temperatures varied only slightly by season or ambient temperature.

For the ILR (and SDD refrigerator; see discussion below), any internal temperature outside the + 2 °C to + 8 °C range was designated an excursion. There were no excursions below + 2 °C in any ILR in either project phase; however, temperature excursions above + 8 °C did occur. The ILR had a 0.4% rate of high-temperature excursions in Phase 1 and 2% in Phase 2. These averaged + 9.6 °C (Phase 1) and + 13.2 °C (Phase 2). There was no difference amongst devices with or without plastic curtains or plastic storage boxes.

The holdover time for the ILR generally exceeded its PQS requirement of 5 days and its rating of 7 to 10 days. In Phase 1, all but one ILR had more than 10 days of holdover time; this ILR had technical issues that resulted in a holdover time of only 1 day. In Phase 2, all but two ILRs had a holdover time of more than 10 days; the exceptions were 6 and 8 days. Using power supply data collected by the project, each ILR received approximately 20 h of electrical supply per day on average in Phase 1 and around 16 h in Phase 2.

All the ILRs malfunctioned at least once during the pilot, especially in Phase 1. The most common reason was failure of the printed circuit board. The voltage stabiliser or compressor also failed occasionally, especially during lightning storms. Some rural areas had power drops and surges considerably beyond the limits of the built-in voltage stabiliser.

In Phase 1, especially during the monsoon season, considerable condensation and black dust accumulation was observed in the ILR, the former causing some damage to the vaccine boxes and vial labels.

#### Solar direct drive refrigerator

Like the ILR, the SDD refrigerator offered reliable protection against freezing: temperatures stayed at greater than + 4 °C at all times. In this assessment, the SDD refrigerator confirmed the WHO PQS Grade A freeze protection rating it earned in laboratory settings.

The mean internal temperature range for the SDD refrigerator was + 4.6 °C to + 6 °C, well within the desired + 2 °C to + 8 °C range (Table 4).

**Table 4:** Internal temperature summary for solar direct drive refrigerators.

**Cold chain point**	**SDD refrigerators—Phase 1** **n = 51,309 data points total**		**SDD refrigerators—Phase 2** **n = 27,184 data points total**	
	**Mean temperature (SD)**	**95% CI**	**Mean temperature (SD)**	**95% CI**
**Bharewala (Rajasthan)**	6.0 (1.19)	6.00–6.04	5.0 (0.44)	(5.01–5.03)
**Motichak (Uttar Pradesh)**	5.2 (0.83)	5.23–5.26	5.0 (0.68)	(4.96–4.99)
**Lumshnong (Meghalaya)**	5.1 (1.21)	5.07–5.12	4.8 (0.62)	(4.78–4.81)
**Chaka (Uttarakhand)**	4.7 (0.24)	4.70–4.71	4.6 (0.39)	(4.55–4.57)

CI: confidence interval; SD: standard deviation; SDD: solar direct drive.

The SDD refrigerator maintained temperatures within the correct range 97.7% of the time in Phase 1 and 99.6% of the time in Phase 2. The mean internal temperature difference in the SDD refrigerator was + 0.1 °C at all ambient temperatures, indicating extreme reliability in maintaining proper temperatures during all seasons.

Although this is a solar device, solar radiation was not measured because the average amount of solar radiation available per day across India in all seasons (3.5 to 4.0 kWh/square meter) is more than the amount required by the SDD refrigerator. The SDD refrigerator experienced no malfunctions.

There were no excursions below + 2 °C in any SDD refrigerator in either project phase. High-temperature excursions did occur, though infrequently. The SDD refrigerator experienced high-temperature excursions 2.3% of the time in Phase 1 and 0.4% in Phase 2. These averaged + 10.6 °C (Phase 1) and + 10.1 °C (Phase 2).

As with the ILR, considerable condensation and black dust accumulation was observed in the SDD refrigerator, the former causing some damage to the vaccine boxes and vial labels.

#### Long-term passive device

The internal temperature range for vaccine storage in the LTPD is greater than 0 °C and less than + 10 °C, as per WHO PQS specifications for passively cooled devices. Internal temperatures less than 0 °C or greater than + 10 °C were considered excursions. The mean internal temperature range for the LTPD was + 0.5 °C to + 2 °C in all seasons (Table5).

**Table 5:** Internal temperature summary for long-term passive devices.

**State**	**Long-term passive devices—Phase 1** **n = 225,381 data points total**		**Long-term passive devices—Phase 2** **n =** 122,662 data points total	
	**Mean temperature (SD)**	**95% CI**	**Mean temperature (SD)**	**95% CI**
**Rajasthan**	0.7 (1.48)	0.68–0.70	0.7 (1.15)	0.71–0.73
**Uttar Pradesh**	2.0 (3.53)	1.91–1.99	0.8 (2.01)	0.81–0.87
**Meghalaya**	0.8 (2.99)	0.77–0.81	0.5 (1.87)	0.49–0.53
**Uttarakhand**	1.0 (2.88)	0.94–0.99	0.7 (2.41)	0.62–0.68

CI: confidence interval; SD: standard deviation.

In Phase 1, the device maintained temperatures within the correct range 95.6% of the time. In Phase 2, the percentage of temperatures in the correct range was 98.1%. The internal temperature in the LTPD, though consistently low, rose very slightly as the ambient temperature rose.

Eight non-standard ice blocks were used in the LTPD to maintain proper storage temperatures, which required conditioning to prevent freezing. The LTPD had low-temperature excursions 2.1% of the time in Phase 1 and 0.9% of the time in Phase 2. Most temperature excursions below 0 °C in both phases were observed after the monthly replacement of ice blocks. When temperatures did drop below 0 °C, the mean internal temperatures were –0.52 °C in Phase 1 and –0.15 °C in Phase 2. Because the LTPD is so efficient at maintaining temperatures, these low temperatures sometimes persisted for up to 9 days. The external temperature display on several of the LTPDs malfunctioned, requiring the manufacturer to replace all solar sensors with a 3.6 V, 900 mAh lithium battery with a life of 5 years. In addition, the alarm, which signals temperature excursions, was not audible to most CCHs.

High-temperature excursions greater than + 10 °C occurred in the LTPD 2.4% of the time in Phase 1 and 1.1% of the time in Phase 2. These averaged + 15.4 °C (Phase 1) and + 15.6 °C (Phase 2). Condensation was seen in a few LTPDs, and the labels on vaccine vials stored in the top box got wet.

### Equipment acceptability

CCHs reported that all three devices were acceptable, specifically noting their reliability. Ease of use was also noted, especially for the ILRs and SDD refrigerators, due to their similarity to a standard refrigerator. The front-door design was appreciated by the CCHs, who felt this improved the visibility and placement of the vaccines inside the equipment compared to a chest refrigerator. Yet condensation was an issue, causing some damage to the vaccine boxes and vial labels unless vaccines were placed inside plastic boxes.

A challenge with the ILRs and SDD refrigerators was location of the small light-emitting diode (LED) alarms, which indicate malfunction, on top of the tall equipment, making them hard to see. This, combined with the lack of an audible alarm, served to reduce the value of the long holdover as health workers noticed equipment malfunction only after the internal temperatures had risen for a few days.

The need to condition ice blocks for the LTPD; difficulty of positioning the last ice block in the small, tight space; and at times, the need to remove all vaccines during immunisation sessions (due to the vaccine stack design) made the LTPD somewhat difficult to operate.

Nonetheless, all health workers agreed on the effectiveness of the three devices in ensuring vaccine potency due to their ability to maintain a uniform temperature range.

### Systems fit

The greatest benefit to the health system of the new cold chain equipment was in helping to establish new CCPs, which reduced the travel time for health workers. Most outreach sites were then within a 1-hour reach of a CCP, meeting a stated goal of the MoHFW. With less time spent travelling, some ANMs reported an increase in on-time vaccination sessions; longer vaccination sessions; and more time to work on other tasks, such as preparing reports, making lists of children due for vaccination, and coordinating with other health personnel. Longer sessions also provided ANMs more opportunity to interact with beneficiaries and discuss the benefits of immunisation with caregivers. For some ANMs, the number of beneficiaries increased as session timing became more regular, raising the possibility of an increase in immunisation coverage.

Many health workers felt they were more efficient and productive with the new equipment. They also appreciated the devices’ efficient use of power, which allowed new CCPs to be established closer to beneficiaries and closer to their own homes. New CCPs also helped address vaccine shortages and stockouts, as ANMs could more easily collect and store additional vaccines. Health workers perceived that fewer vaccines were wasted due to the long holdover times of all three devices and because it became easier to return vaccines to the CCP at the end of a session or after a long travel day.

#### Device limitations

A key limitation of all three devices was the inability to freeze ice packs, which are required for immunisation outreach. In facilities with limited or no power, the value of the LTPD was also diminished by the need to freeze its eight ice blocks. The limited storage capacity of the LTPD was an additional disadvantage, primarily during vaccination campaigns and in areas catering to a larger population (e.g., Uttar Pradesh).

For the LTPD, removing vaccines often required removal of the entire vaccine stack, which was inconvenient and may have exposed vaccines to more time out of the cold chain.

#### Other limitations

Human resources were inadequate in some project areas; for example, in several locations, ANMs also served as CCHs, reducing the time they had to provide immunisation services. In addition, alternative vaccine delivery (AVD) personnel (mandated by the Indian government) were not always available.

Some new CCPs did not receive adequate vaccine supply due to stock shortages at the parent CCP. This necessitated the CCHs to make multiple visits to the parent CCP at higher personal cost and greater inconvenience, which diminished the benefit of having a new CCP.

### Costs

Costs are presented in both US dollars (US$) and Indian rupees (INR), using average 2017 exchange rates (US$1 = 67 INR).

#### Installation

All costs related to installation of the ILRs and SDD refrigerators, including transport for the installation team and equipment, were borne by the manufacturer and included in the equipment purchase price. Each installation cost US$200 (13,413 INR). When deep freezers were required and provided by the project, this one-time transport cost was US$112 (7,511 INR).

#### ***Modifications and*** repairs

Because both the second-generation ILR and SDD refrigerator are front opening devices, hot air rushed into the devices whenever the doors were open, causing the temperature to increase and condensation to develop. To address this issue, the manufacturer provided plastic strip curtains, which were installed in all ILRs and SDD refrigerators, at a cost of US$21 (1,408 INR) per refrigerator. In addition, vaccines had to be kept in plastic containers, at a cost of US$7 (469 INR) per refrigerator. Thus, there was an additional one-time cost of approximately US$30 (2,012 INR) per ILR and SDD refrigerator. This excluded the labour and transport costs of the technicians who installed the curtains at each CCP.

The main malfunction reported for the ILRs was failure of the printed circuit boards. These were replaced at US$27 (1,810 INR) per circuit board. For the LTPDs, the solar display panels were replaced with lithium batteries at US$21 (1,408 INR) per device. All repairs and equipment modifications were done by technicians employed by the manufacturers, and the costs were borne by the manufacturers.

#### Trainings

Start-up training costs per CCH were estimated at US$77 (5,164 INR). In addition, the CCHs requested refresher training, training on maintenance and cleaning of the CCP, and training on early identification of equipment malfunction.

#### Cold chain points

We estimated the total average annual costs for human resources, equipment, and transport for each new CCP at US$9,985 (669,004 INR).

Salaries for CCP staff, who were already being paid by the MoHFW, comprised by far the largest share of this cost (Table 6). Yet because salary costs reflected current staffing levels rather than ideal staffing levels as defined by government policy, they may have been underestimated. Across the pilot project, the time spent preparing for outreach sessions varied from 0.5 to 2.5 h, depending on the number of sessions, and the time spent tracking and placing usable vaccines back into the cold chain equipment after the sessions ranged from 1 to 2 h.

**Table 6:** Estimated average annual costs of equipment, transportation, and human resources per cold chain point.

**Cost category**	**Average annual costs** **per CCP (INR)**	**Average annual costs** **per CCP (US$)**
Average cold chain costs per CCP		
Cold chain equipment depreciation	20,657	308
Cold chain equipment energy costs	2,294	34
Generator costs	17,032	254
Total cold chain costs	39,983	597
Average transport costs per CCP		
Vaccine collection	14,466	216
Outreach	5,039	75
Alternative vaccine delivery costs	18,600	278
Ice blocks for long-term passive devices	3,126	47
Total transport costs	41,232	615
Average human resources cost per CCP (salaries)	587,789	8,773
Annual average total cost per CCP	669,004	9,985

CCP: cold chain point; INR: Indian rupee; US$: United States dollar.

For equipment, we annualised the purchase price using the straight-line method and assuming a 10-year useful life. Estimated cold chain costs included new CCP equipment and freezers for freezing ice packs, as neither the ILR, SDD refrigerator, nor LTPD can freeze ice packs—yet these are required for outreach sessions, where most vaccinations in India are delivered.

CCPs without a deep freezer reported spending between US$6 (402 INR) and US$32 (2,146 INR) per month collecting ice blocks for the LTPD from nearby facilities. This translated to an annual cost of US$47 (3,126 INR) per facility per year when averaged across all facilities, or US$177 (11,870 INR) when including only the CCPs that reported these costs.

At baseline, all existing CCPs were using ILRs, and about half of CCPs had a generator for back-up power. The monthly costs of running generators varied greatly by district, from US$4.50 (302 INR) to US$270 (18,107 INR) per month, likely due to the erratic electrical supply.

#### Cost per storage capacity

We calculated the capital and energy cost per litre of storage for each device as the purchase price, including spare parts and installation costs, and the energy cost over the useful life (10 years) divided by the vaccine storage capacity [15]. These costs were US$31 (2,079 INR) for the ILR, US$75 (5,039 INR) for the SDD refrigerator, and US$526 (35,276 INR) for the LTPD (Table7). For the LTPD, these figures do not include the cost of freezing ice blocks or transporting ice packs if the freezing was done at another facility. Including these costs for the LTPD increases the cost per litre to US$612 (41,027 INR). In addition, storage of the vaccines in plastic boxes resulted in a reduction in net vaccine storage volume for the ILRs and SDD refrigerators; the estimated cost per litre of storage would be higher than reported if plastic boxes were used as a long-term solution.

**Table 7:** Costs and storage capacity of cold chain equipment installed.

	**Second-generation ice-lined refrigerator (INR)**	**Second-generation ice-lined refrigerator (US$)**	**Solar direct drive refrigerator (INR)**	**Solar direct drive refrigerator (US$)**	**Long-term passive** **device (INR)**	**Long-term passive** **device (US$)**
Purchase price	179,532	2,677	422,038	6,293	155,120	2,313
Capital expenditure (purchase price; spare parts and installation costs)	195,292	2,912	495,138	7,383	190,463	2,840
Energy costs over a 10-year useful life	1,274	19	0	0	0	0
Transport costs for ice collection over a 10-year period	Not applicable	Not applicable	Not applicable	Not applicable	31,263	467
Vaccine storage capacity (litres)	99	99	99	99	5.4	5.4
Capital and energy cost per litre of storage, excluding costs of ice collection, if applicable to the equipment	2,079	31	5,039	75	35,276	526
Capital and energy cost per litre of storage, including costs of ice collection, if applicable to the equipment	2,079	31	5,039	75	41,027	612

#### Costs ***for immunisation outreach***

At baseline, ANMs reported a wide range of distances to session sites, from 1 to 150 km, which took 0.25 to 5 h to traverse. Toward the end of the study, they reported notably shorter distances travelled, ranging from 4 to 33 km. ANMs generally used public transport to visit session sites. Others walked. The costs for ANMs to travel were not included in our analysis as they were out-of-pocket expenditures. The largest share of the transport costs was for AVD services. In Uttar Pradesh and Uttarakhand, where the AVD system is well established, AVD personnel were paid US$1.15 (77 INR) and US$2.30 (154 INR), respectively, to transport vaccine carriers.

#### Vaccine wastage

Due to inadequate facility records, we were unable to collect facility-level data on vaccine inventory. This prevented analysis of the effects of the intervention on vaccine availability and usage. Whereas vaccine stock data were uploaded to the block level in the health management information system portal, facility-level data were manually recorded in stock registers at each facility, and at some facilities, the data were inconsistently collected or incomplete. Nonetheless, some CCHs felt the strong performance of the piloted cold chain equipment may have reduced closed-vial vaccine wastage.

## Discussion

The three devices evaluated during the pilot project (second-generation ILR, SDD refrigerator, and LTPD) were different from each other in terms of technology, specifications, and performance so are not directly comparable. The SDD refrigerator demonstrated absolute technical reliability throughout the pilot. Despite its high up-front cost, its consistent performance in all climate and environmental conditions makes it a promising option for facilities that are difficult to reach and have unreliable power. As noted previously, there were no excursions below + 2 °C in any SDD refrigerator in either project phase; however, high-temperature excursions did occur, though infrequently. As there were no reported device malfunctions amongst the SDD refrigerators, it is possible the refrigerator doors were left open for too long, causing temperatures to rise.

The ILR experienced difficulties in settings with uneven power supply. The voltage stabiliser cut off at 140 V, causing the circuit board to short-circuit and fuse to protect the equipment. The manufacturer addressed this issue early on by providing an external voltage stabiliser with a range of 110 to 280 V, and in the future intends to equip all ILRs for a wider voltage range. Given the relatively small number of devices included in the pilot, there was adequate time to make these repairs. However, during a wider-scale deployment, it would be important to consider how repairs would be coordinated and implemented and whether the manufacturers would again bear the costs of parts, transport, and labour. There were no excursions below + 2 °C in any ILR in either project phase; however, high-temperature excursions did occur during actual use in Phase 2. As with the SDD refrigerator, it is likely the door to the ILR was opened more frequently.

The long holdovers of both the ILR and SDD refrigerator eased health workers’ worries about vaccine potency. For the ILR in particular, since it is reliant on grid electricity, the ability to maintain temperatures for extended periods is critical, especially in light of frequent power cuts, and in some cases, extended power outages. Yet because of the inaudible alarm and the placement of the small LED visual alarm on top of the tall equipment, health workers were often unaware there was a problem until the internal temperature had exceeded + 8 °C, by which point much of the extended holdover time had passed. The ILR would benefit from an audible alarm system. Both the ILR and SDD refrigerator were easy for health workers to learn to use, as they are very similar to a standard refrigerator. Also, the 99-litre capacity of these devices would be sufficient for the larger populations living in much of India.

The challenge of condensation, especially during months of high humidity, needs to be addressed in both devices, as well as the requirement to freeze ice packs for outreach sessions. Measuring condensation is technically challenging, which made it difficult to quantitatively determine any effect the strip curtains had on condensation levels. Manufacturers are encouraged to address the condensation issues, thus ensuring a more hygienic vaccine storage environment. Some reduction of condensation was noticed in Phase 2, but this may have been related to the project concluding in the summer, before the monsoon season.

The LTPD was valued in settings with smaller populations and unreliable or no power. This device may facilitate the creation of new CCPs in areas that can ensure ice block supply and replacement every 35 days. District hospitals and urban health facilities that provide fixed immunisation services may find the LTPD useful for vaccine storage. It should be tested in densely populated areas such as urban slums. In this pilot study, the external temperature display on several of the LTPDs malfunctioned, requiring the manufacturer to replace all solar sensors with a lithium battery with a life of 5 years. It will be important to plan for replacement of these batteries.

The most significant finding related to the LTPDs’ performance was the effect on internal temperatures when ice blocks were placed in the device before being fully conditioned. Hands-on training on how to condition the ice blocks and the addition of an improved ice block temperature dial and alarm system are advised to prevent internal temperatures falling below 0 °C. Although the ice blocks have a dial showing when the ice has reached 0 °C, the accuracy of the visual feature may not be specific enough, or this visual guidance was not always followed by health workers. In both phases, the low-temperature excursions occurred in slightly less than half of the LTPDs.

High-temperature excursions occurred in the LTPD less than 2.5% of the time in both phases. Though not directly observed, it appears likely that high- and low-temperature excursions may have been related to user error. The slight decrease in the number of temperature excursions between Phases 1 and 2 may indicate the value of refresher training and/or the CCHs becoming more comfortable with the LTPD over time.

As per manufacturer instructions, the requirement for the LTPD’s lid to be open no more than 10 min per week was unrealistic, especially during campaigns and outreach sessions. The manufacturer is encouraged to reconsider this requirement, as the devices were able to maintain temperatures within range most of the time. Condensation was also noted in a few LTPDs, usually after the lid had been opened for a session.

The LTPD’s 5.4-litre storage capacity and the need for monthly transport of ice blocks were limitations within the Indian context and must be carefully considered to determine the device’s best use case. To support the introduction of LTPDs, a WHO policy document is needed to articulate the scientific basis for the acceptability of storing vaccines in passively cooled equipment at 0 °C to + 10 °C rather than the longstanding recommendation of + 2 °C to + 8 °C. In these piloted Indian settings—and in other settings where outreach is conducted—it would be optimal to have a combination refrigerator/freezer that is solar powered or requires low power. Manufacturers are strongly advised to consider designing future equipment to allow freezing of ice packs. Ice packs play a crucial role in maintaining appropriate temperatures in vaccine carriers used to conduct immunisation outreach. As most new CCPs had been recently established, an additional deep freezer was necessary to freeze the ice packs. To obtain frozen ice blocks for the LTPD, CCHs had to depend on the adjoining or parent CCP to transport ice blocks each month. Reduction in distance and travel time between the CCPs and session sites may have reduced both closed- and open-vial vaccine wastage due to the ability to return vials to the CCP the same day. Collecting wastage data, which is not currently done, would allow quantitative analysis of this potential benefit.

Regarding cost, the annual capital and energy costs for the LTPD are high because of the small volume of the equipment relative to the purchase price. In the pilot project, replacement of ice packs was done on a different cycle from replenishment of vaccines; for maximum efficiency, these tasks should be combined if possible.

Based on project findings, the qualitative criteria shown in Table 8 may assist decision-makers with selection and deployment of the ILRs, SDD refrigerators, and LTPDs. Each criterion was given equal weight. For settings outside India, criteria can be weighted to give a more accurate value.

**Table 8:** Considerations for deployment of the second-generation ice-lined refrigerator, solar direct drive refrigerator, and long-term passive device in India. ey: ○ Yes, good Δ Moderate, somewhat □ No, relatively high cost.

Abbreviations: ILR, ice-lined refrigerator; LTPD, long-term passive device; PQS, Performance, Quality and Safety; SDD, solar direct drive; WHO, World Health Organisation. *Godrej & Boyce MFG. Co. Ltd., GVR 100 AC (Sure Chill®); †Godrej & Boyce MFG. Co. Ltd., GVR 100 DC (Sure Chill®); ‡AUCMA C0. Ltd, ARKTEK™ model YBC-5 (P6).

## Conclusions

The introduction of new cold chain technologies in India is intended to ensure the equitable and effective delivery of vaccines to all children in the country, no matter where they live. This project demonstrated the performance, acceptability, systems fit, and costs and benefits of the three cold chain technologies in addressing the challenges of India’s immunisation system. The project focused on the challenges of temperature extremes, difficult-to-reach terrain, and erratic or limited power. The results highlight the need for technologies and systems strengthening efforts to be tailored to the diverse conditions in India’s cold chain. This includes in rural areas, where 72% of the country’s population resides [16]. This pilot test demonstrated the robustness of three cold chain technologies and the criteria for which they would each add the most value. The data for the three devices showed reliable temperature performance and that the devices were consistent at maintaining temperatures*.* CCHs and ANMs appreciated the ease of use, reliability, and simplicity of these devices and, importantly, appreciated the new CCPs these technologies made possible. All three devices performed well, although all of the ILRs experienced at least one failure, most often the circuit board, but also the voltage stabiliser or compressor.

An important design limitation of each device was the inability to freeze ice, which is necessary for immunisation outreach with vaccine carriers. The requirement to collect ice from the closest powered CCP somewhat limits the potential these technologies have in reaching the people who are hardest to reach. This pilot study points to the value of a combination refrigerator/freezer that can operate on low or solar power.

The Indian government will ultimately decide which equipment to purchase, but the results of this study may help inform their decision-making process.

## Declaration of Competing Interest

The authors declare that they have no known competing financial interests or personal relationships that could have appeared to influence the work reported in this paper.

## Data Availability

The data are included in the article.
